# Student’s experiences with online teaching following COVID-19 lockdown: A mixed methods explorative study

**DOI:** 10.1371/journal.pone.0250378

**Published:** 2021-08-31

**Authors:** Kari Almendingen, Marianne Sandsmark Morseth, Eli Gjølstad, Asgeir Brevik, Christine Tørris

**Affiliations:** 1 Faculty of Health Sciences, Department of Nursing and Health Promotion, Oslo Metropolitan University, Oslo, Norway; 2 Department of Primary and Secondary Teacher Education, Faculty of Education and International Studies, Oslo Metropolitan University, Oslo, Norway; KTH Royal Institute of Technology, SWEDEN

## Abstract

**Background:**

The COVID-19 pandemic lead to a sudden shift to online teaching and restricted campus access.

**Aim:**

To assess how university students experienced the sudden shift to online teaching after closure of campus due to the COVID-19 pandemic.

**Material and methods:**

Students in Public Health Nutrition answered questionnaires two and 12 weeks (N = 79: response rate 20.3% and 26.6%, respectively) after the lockdown in Norway on 12 March 2020 and participated in digital focus group interviews in May 2020 (mixed methods study).

**Findings and discussion:**

Two weeks into the lockdown, 75% of students reported that their life had become more difficult and 50% felt that learning outcomes would be harder to achieve due to the sudden shift to online education. Twelve weeks into the lockdown, the corresponding numbers were 57% and 71%, respectively. The most pressing concerns among students were a lack of social interaction, housing situations that were unfit for home office purposes, including insufficient data bandwidth, and an overall sense of reduced motivation and effort. The students collaborated well in digital groups but wanted smaller groups with students they knew rather than being randomly assigned to groups. Most students agreed that pre-recorded and streamed lectures, frequent virtual meetings and student response systems could improve learning outcomes in future digital courses. The preference for written home exams over online versions of previous on-campus exams was likely influenced by student’s familiarity with the former. The dropout rate remained unchanged compared to previous years.

**Conclusion:**

The sudden shift to digital teaching was challenging for students, but it appears that they adapted quickly to the new situation. **A**lthough the concerns described by students in this study may only be representative for the period right after campus lockdown, the study provide the student perspective on a unique period of time in higher education.

## Introduction

The Coronavirus 2019 (COVID-19) pandemic has caused extraordinary challenges in the global education sector [1,2]. Most countries temporarily closed educational institutions in an attempt to contain the spread of the virus and reduce infections [3]. In Norway, the move to online teaching and learning methods accelerated as a consequence of the physical closure of universities and university colleges on 12 March 2020 [4]. Education is better implemented through active, student-centered learning strategies, as opposed to traditional educator-centered pedagogies [5,6]. At the time of the COVID-19 outbreak, the decision to boost the use of active student-centered learning methods and digitalisation had already been made at both the governmental and institutional levels [7,8] because student-active learning (such as use of student response systems and flipping the classroom) increase motivation and improve learning outcomes [5,7,9]. However, the implementation of this insight was lagging behind. Traditional educator-centered pedagogies dominated higher education in Norway prior to the lockdown, and only 30% of academic teachers from higher institutions reported having any previous experience with online teaching [4]. Due to the COVID-19 lockdown, most educators had to change their approaches to most aspects of their work overnight: teaching, assessment, supervision, research, service and engagement [4,10].

Bachelor’s and master’s in Public Health Nutrition (PHN) represents two small-sized programmes at Oslo Metropolitan University (OsloMet). PHN is defined as ‘the application of nutrition and public health principles to design programs, systems, policies, and environments that aims to improve or maintain the optimal health of populations and targeted groups’ [11,12]. Traditional teaching methods dominated on both programs during winter 2020. Following the lockdown, online learning for the continuation of academic activities and the prevention of dropouts from study programmes in higher education were given the highest priority. Due to an extraordinary effort by both the administrative and academic staff, digital alternatives to the scheduled on-campus academic activities were offered to PHN students already in the first week following lockdown. The scheduled on-campus lectures were mainly offered as live-streamed plenary lectures lasting 30–45 minutes, mainly using the video conferencing tool Zoom. Throughout the spring semester educators received training in digital teaching from the institution and increasingly made use of online student response systems (such as Padlet and Mentimeter) as well as tools to facilitate digital group-work (Zoom/Microsoft Teams). Non-theoretical lectures (e.g. cooking classes), were cancelled, and face-to-face exams were re-organized into digital alternatives in order to ensure normal teaching operations. Several small tweaks were employed to minimize dropout. There was no time for coordinating the different courses with regards to the types of online teaching activities, exams and assessments. Social media, i.e Facebook, and SMS were the primary communication channels the first week after lockdown. The use of learning management systems (LMS) Canvas and digital assessment system, Inspera, remained mainly unchanged. Due to the new situation, the deadline for the submission of bachelor theses was postponed by 48 hours. In addition, bachelor students submitting their thesis where given permission to use the submission deadline for the deferred exam in August as their ordinary exam deadline. The deadline for the submission of master theses was extended by one week, but all planned master exams were completed by the end of June, including oral examinations using Zoom instead of the traditional face-to-face examinations on campus. Even though most of the new online activities where put in place with limited regard for subtle nuances of pedagogical theory, and did not allow for much student involvement, the dropout rate from PHN programs remained unchanged compared to previous years. PHN is a small-sized education with close follow up of students. However, although the students experienced a digital revolution overnight, we know little about how they experienced the situation after the university closed for on-campus activities.

Accordingly, the purpose of this study was to assess how Norwegian PHN students experienced the shift to digital teaching following campus lockdown. Students were also asked to provide feedback on what might improve the learning outcomes in future online lectures and courses.

## Methods

### Design and sampling

This study utilised a mixed methods cross-sectional design, where quantitative and qualitative methods complemented each other. An invitation to participate was sent out to 79 eligible students via multiple channels (Facebook, Teams, Zoom, LMS Canvas, SMS), with several reminders. The only eligibility criteria was being a student in PHN during spring 2020. All students received the quantitative survey. Due to few students eligible for each focus group interview, all who wanted to participate were interviewed/included. The invited students were in their second-year (n = 17) and third-year (n = 28) bachelor’s and first-year (n = 13) and second-year (n = 21) master’s programme at PHN in the Faculty of Health Sciences at OsloMet. The response rate was 16/79 (20.3%) and 21/79 (26.6%). Two focus group interviews were scheduled in each class (a total of 8) but only 4 interviews were conducted. The research team was heterogeneously composed of members with both pedagogical and health professional backgrounds.

### Online questionnaire

To the best of our knowledge, this study was the first “corona” study at our Faculty. No suitable national or international questionnaire had been developed and /or validated by March 2020. Hence, online questionnaires for the present study were designed virtually ‘over-night’. The questions were however based on experiences from a large-scale interprofessional learning course using the blended learning approach at OsloMet [13,14] and specific experiences that academic staff in Norway reported during the first week of teaching during the lockdown [4]. The questionnaires were based on an anonymous self-administrated web survey ‘Nettskjema’ [15]. ‘Nettskjema’ is a Norwegian tool for designing and conducting online surveys with features that are customised for research purposes. It is easy to use, and the respondents can submit answers from a browser on a computer, mobile phone or tablet. During the first week after lockdown, the questionnaire was sent out to university colleagues and head of studies and revised accordingly. The questionnaires were deliberately kept short because the response rate is generally low in student surveys [16]. Ideally, we should have pretested and validated the questionnaires, but this was not possible within the short-time frame after lockdown. Items were measured on a five-level ordinal scale (Likert scale 0–5). The two forms contained both numerical and open questions, permitting both quantitative and qualitative analyses. The first questionnaire was sent out to the students on 25 March 2020 (two weeks after the closure of university campus; students were asked to submit their answers during the period from 12 March until the link was closed at Easter Holiday), and the second questionnaire was sent on 3 June 2020 (12 weeks after closure; students were asked to submit their answers during the period after Easter and until the end of the spring semester). The questionnaires were distributed as web links embedded in the LMS Canvas application. Because live-streamed lectures were offered primarily through Zoom during the first weeks, students were not asked about interactive digital teaching and tools in the first questionnaire. At the end of both questionnaires, the students were asked what they believed could improve the learning experience in future online education. The qualitative part consisted of text answers to open questions from the two electronic questionnaires.

### Digital focus group interview

To capture meaningful insights into the participants experiences, we conducted digital focus group interviews [17], aiming to conduct one digital focus group interview in each class. PHN is a small sized education, and the teachers know all the students. The focus group interviews were therefore performed by two external independent researchers (EG and CT) who are not directly involved in the PHN education and had no prior knowledge to the students. The two interviewers (moderators) were middle-aged female teachers working in the university, and both have significant experience in digitalizing education. They were presented to the participants as researchers from the university. The report of this study was guided by the consolidated criteria for reporting qualitative research (COREQ). The interviews were conducted via the video conferencing system Zoom during May 2020, following internal guidelines [18]. In the focus group interviews, the participants reflected on their own experiences, and the moderator guided the discussion using a semi-structured interview guide. This guide was prepared based on the research questions. One pilot interview was conducted, which resulted in some minor changes to the interview guide. The results from the pilot interview are not included in the results. The focus group interviews lasted for approximately one hour, and five students were invited to each focus group interview. The interviews were not recorded, but the moderator took notes, ensuring that the participants remained anonymised.

### Data analysis

Quantitative data are described descriptively with numbers and percentages. Apart from re-categorization of response categories, no statistical analysis was performed. Quantitative data were extracted directly from the survey system. Answers in categories 0 or 1 were categorised as ‘Disagree/slightly agree’, answers in categories 2 or 3 were categorised as ‘Somewhat agree’ and answers in categories 4 or 5 were categorised as ‘Agree’. Qualitative data were analysed using systematic text condensation (STC), inspired by Giorgi’s phenomenological approach and modified by Malterud [17]. First, the entire texts (from the interviews) were read to get an overall impression, and preliminary themes were derived from the interviews. Then, meaning units, such as sentences and words, were identified and connected with the preliminary theme to elucidate the study question. The meaning units were then coded and systemized into groups, so that meaning could be abstracted from the different code groups. Finally, the meanings of the various units were summarised. The qualitative data from the questionnaire were then extracted by the moderators, and the words and sentences were identified and abstracted. In order to ensure quality, the notes from the focus group interviews and the text answers from the questionnaires were reviewed by both moderators.

### Ethical considerations

All participants gave their informed consent. The questionnaires did not include questions about personal health information or sensitive data. The quantitative data were collected through an anonymous web survey using ‘Nettskjema’ [15]. Internal routines at OsloMet for using Zoom in research interviews were applied [18]. In the interviews, the participants provided their written consent in the chat without their names and remained anonymous. The data protection was approved by the Norwegian Centre for Research Data (NSD, reference no. 846363), as PHN is a small-sized study programme and because Zoom was used for the digital focus group interviews.

## Results

### Quantitative data

There were 16 (20.3%) and 21 (26.6%) students who answered the questionnaires two and 12 weeks after lockdown, respectively (Table 1). Both samples had an even distribution of bachelor and master students.

**Table 1:** Characteristics of the sample and views about studying, two and 12 weeks after the national lockdown in Norway on 12 March 2020 due to the COVID-19 pandemic.

	2 weeks after lockdown n = 16	12 weeks after lockdown n = 21
	n (%)	n (%)
Age, years		
≤ 21	0 (0)	0 (0)
22–25	7 (44)	11 (52)
26	9 (56)	10 (48)
Study programme		
Bachelor	8 (50)	11 (52)
Master	8 (50)	10 (48)
Prior experience with digital learning	7 (44)	9 (43)
Expectations upon learning outcome from digital education		
Higher	3 (19)	0 (0)
Lower	8 (50)	15 (71)
No change	6 (38)	5 (24)
Do not know ^a^	NA	2 (10)
Studying has become more challenging after COVID-19 lockdown	12 (75)	12 (57)

^a^ Only after 12 weeks.

Among the respondents two and 12 weeks after lockdown, 7/16 students (44%) and 9/21 students (43%) reported having previous experience with online learning, respectively (Table 1). After two weeks of forced online education, 8/16 students (50%) expected that their learning outcomes would be inferior with online education compared to their pre-COVID-19 education at campus. After 12 weeks, 15/ 21 students (71%) expected that their learning outcome would be lower, and, notably, none of the students expected that it would be higher. On both occasions, most students reported that studying had become more difficult compared to the time before the pandemic.

Several of the identified challenges with online education were reported by more than 50% of the students, and there was an uneven spread across categories of answers (Tables 2 and 3). Only one of 16 students (6%) agreed that they needed to increase their digital competence, but approximately half reported having technical challenges at home. All of the students agreed that the lack of contact with other students was a challenge. However, after 12 weeks, the lack of contact with academic staff seemed to pose less of a challenge.

**Table 2:** Challenges with digital education two weeks after the COVID-19 lockdown (sample before Easter, n = 16).

Challenge	Disagree/slightly agree^a^ n (%)	Somewhat agree^b^ n (%)	Agree^c^ n (%)
Cannot study due to changes in society	4 (25)	8 (50)	4 (25)
Technical: computer at home	10 (63)	3 (19)	3 (19)
Technical: lack of digital competence	13 (81)	2 (13)	1 (6)
Lack of social interaction	0 (0)	3 (20)	13 (80)
Work situation (children, home)	6 (38)	8 (50)	2 (13)
Privacy issues	12 (75)	3 (19)	1 (6)
Sickness	12 (75)	1 (6)	3 (19)

^a^ On a scale from 0 to 5, answers 0 or 1.

^b^ Answers 2 or 3.

^c^ Answers 4 or 5.

**Table 3:** Challenges with digital education 12 weeks after the COVID-19 lockdown (sample at end of semester, n = 21).

Challenge	Disagree/slightly agree^a^ n (%)	Somewhat agree^b^ n (%)	Agree^c^ n (%)
Technical: computers	10 (48)	6 (29)	5 (24)
Technical: insufficient bandwidth	12 (58)	5 (24)	4 (19)
House unfit for home office purposes	5 (24)	3 (14)	13 (62)
Lack of academic contact with peers	1 (5)	1 (5)	19 (91)
Lack of academic contact with staff	3 (14)	9 (43)	9 (43)
Lack of social contact with peers	0 (0)	4 (19)	17 (81)
Reduced motivation and effort	1 (5)	11 (52)	9 (43)
Lower learning outcome	5 (24)	7 (33)	9 (43)
Inadequate information about exam	3 (14)	11 (52)	7 (33)
Desire to return to on campus activates	0 (0)	2 (10)	19 (91)

After 12 weeks, 20/21 students (95%) agreed that their motivation and effort had been reduced. At the same time, all students wanted to return to campus. Only 5/21 (24%) reported that their learning outcomes had not deteriorated.

### Suggestions for how to increase learning outcome in future digital courses

Two weeks after lockdown, most students answered that the use of different components of online education would improve the learning outcomes in a future online course (Table 4). Regarding participation in digital group work, there was a nearly even spread across the different categories of answers. Finally, participants preferred written home exams and feedback over the digital options suggested (Table 5).

**Table 4:** Activities that would increase learning outcomes in future digital courses (sample before Easter, n = 16).

Activity	Disagree/slightly agree^a^ n (%)	Somewhat agree^b^ n (%)	Agree^c^ n (%)
Podcast	3 (19)	5 (31)	8 (50)
Recorded in advance	0 (0)	4 (25)	12 (75)
Live streaming	2 (13)	4 (25)	10 (62)
Virtual seminars	2 (13)	4 (25)	10 (62)
Participation in chats	2 (13)	6 (38)	8 (50)
Participation in digital student groups	5 (31)	4 (25)	7 (44)
Increased number of written submissions	4 (25)	7 (44)	5 (31)
Increased use of student response systems (Kahoot, Mentimeter etc)	3 (19)	6 (38)	7 (44)
Increased degree of mandatory participation	5 (31)	9 (56)	2 (13)

^a^ Answered 0 or 1 on a scale from 0 to 5.

^b^ Answered 2 or 3.

^c^ Answered 4 or 5.

**Table 5:** Types of exams that would be suitable in a future digital course (sample before Easter, n = 16).

Type of exam	Disagree/slightly agree^a^ n (%)	Somewhat agree^b^ n (%)	Agree^c^ n (%)
Multiple choice	3 (19)	4 (25)	9 (56)
Written home exams	0 (0)	3 (19)	13 (81)
Video recordings (submissions)	9 (56)	3 (19)	4 (25)
Podcast (submissions)	11 (67)	2 (13)	3 (19)
Live exam via Zoom/Skype/teams etc.	2 (13)	7 (44)	7 (44)
Written feedback	0 (0)	4 (25)	12 (75)
Oral feedback	4 (25)	8 (50)	4 (25)

^a^ On a scale from 0 to 5, answers 0 or 1.

^b^ Answers 2 or 3.

^c^ Answers 4 or 5.

After 12 weeks of (forced) online teaching, more ambivalence toward the use of digital learning tools could be detected (Table 6). However, the proportion of students who agreed that digital group work would increase the learning outcomes seemed unchanged (around 1/3 of both samples). In line with the findings obtained only two weeks after lockdown, written submissions and feedback seemed to be preferable to digital exam options (Table 7).

**Table 6:** Activities which would increase learning outcomes in future online courses (sample at end of semester, n = 21).

Activity	Disagree/slightly agree^a^ n (%)	Somewhat agree^b^ n (%)	Agree^c^ n (%)
Participate in digital student groups^a^	7 (33)	12 (57)	2 (10)
Flipped classroom^a^	8 (38)	11 (52)	2 (10)
Student response systems (Kahoot, Mentimeter etc)	3 (14)	9 (43)	9 (43)
Written submission of assignment	3 (14)	7 (33)	11 (52)
Digital submission of assignment (podcast, film etc)	8 (38)	7 (33)	6 (29)
Frequent digital meeting with academic staff	2 (10)	7 (33)	12 (57)

^a^ Instead of seeing lectures live/streamed lectures?.

**Table 7:** Forms of exams that would be suitable in a future digital course (sample at end of semester, n = 21).

Type of exam	Disagree/slightly agree^a^ n (%)	Somewhat agree^b^ n (%)	Agree^c^ n (%)
Multiple choice	6 (29)	5 (24)	10 (48)
Written home exam	0 (0)	8 (38)	13 (62)
Digital recordings (submission of video/podcast)	11 (52)	7 (33)	3 (14)
Live exams via Zoom/Skype/teams etc.	9 (43)	8 (38)	4 (19)
Written feedback	1 (5)	5 (24)	15 (71)
Oral feedback	4 (19)	5 (24)	12 (57)

After 12 weeks, 16/21 students (76%) agreed that social interaction plays a role in learning outcomes and well-being (Table 8), and an equal proportion agreed that it was important that everyone had their camera on during teaching.

**Table 8:** Statements relevant to online education (sample at end of semester, n = 21).

Statement	Disagree/slightly agree^a^ n (%)	Somewhat agree^b^ n (%)	Agree^c^ n (%)
Social interaction does not influence learning outcome and satisfaction	16 (76)	3 (14)	2 (10)
It is important that everybody has camera on (video on)	5 (24)	6 (28)	10 (48)
It is important that the grouping is random so that everyone can get to know each other	8 (38)	7 (33)	6 (29)
My digital skills have increased	6 (29)	11 (52)	4 (19)

There were 15/21 students (71%) who agreed that their digital competence and interest in digital teaching methods had increased while 6/21 students (29%) disagreed with this statement.

### Qualitative data

In total, there were four master students who participated in digital focus group interviews (on two different occasions, with three students and one student in the groups, respectively).

#### Digital lectures

The students were satisfied with the teaching and reported that the lecturers were competent in arranging online teaching. The lecturers were also good at adapting to the students’ wishes regarding teaching. Lectures that were streamed live (synchronous classes) were preferred over recordings (asynchronous). One student said it was a privilege to still be able to study even though the university campus was closed due to corona and all the lectures were digital. The students expressed that it is an advantage if the lecturer has digital competence to ensure that the lecture runs smoothly without digital/technical problems, or if there is a co-host who can assist. Technical competence is also important when invitation links are sent out. It signals that the student group is well taken care of. The informants described a course co-ordinator as a person with a good overview and sense of responsibility—someone who is good at structure and order. These qualities were highlighted as important in a fully digitalised teaching program.

The students did not support compulsory attendance, as it would reduce the feeling of freedom that most students value. If learning activities were compulsory, students felt it might also present challenges in dealing with their children and part-time work. The students expressed that most of their fellow students were present in lectures that went live on Zoom. One student stated that live digital lectures were best because it was easier to ask questions. When using a flipped classroom or recordings, the questions must be written down and asked afterwards, but both options (flipped classroom and live streaming) were perceived as fine.

Interestingly, the qualitative results from the questionnaire indicated that some students found it easy to ask questions, while others thought it had become more difficult. According to one student, ‘As long as we have the opportunity to ask questions online, I think it will go just fine. I commute three hours per school day to get to and from school, so I feel I have more time to work with school now that the lecture is online’.

One of the informants thought that interaction was challenging, and it did not feel as natural to ask questions in online classes. ‘Raising your hand’ was not perceived to be as easy as in the face-to-face setting on campus, which could mean that the students did not always get answers to their questions.

The students’ indicated that recorded lectures should not be longer than one hour, as it is easy to lose focus, and one must rewind the recordings. For live online lectures, two hours was deemed fine, and they were perceived as fun to watch. However, each session of the live online lectures should not be longer than 45 minutes.

The online teaching (mainly in the form of synchronous plenum lectures originally intended as on-campus lectures) was challenging in the beginning because some students fell out of the digital rooms due to technical reasons, but it got better over time. Some students experienced poor bandwidth, which led to them not being able to turn on their camera and reduced sound quality. One student stated that poor internet quality was something he could not do anything about, but it resulted in a non-optimal learning situation. It was suggested that using a flipped classroom/recorded lectures in the first weeks after lockdown could have solved this problem.

The respondents pointed out that the use of several conference systems/channels in addition to LMS Canvas provided a poor overview and ineffective communication, and they would prefer a single learning platform. The students were unsure how to contact their teachers in the first weeks after lockdown due to the use of several platforms. Even with a single contact channel (LMS), the students found that the threshold barrier for sending questions to the teacher through email was high.

When asked what they thought about ‘black screens’ (students turning off the camera), several answered that this reduced the quality of communication between the lecturer and student. The lecturer missed affirmative nods from students, and the students also likely missed parts of the communication when the camera was turned off. In some of the lectures, all of the students were encouraged to keep the camera on, and some of the lecturers asked the students questions to initiate two-way communication. The students expressed that it was nice to see the other attending students on video. Furthermore, the participants felt that the lecturers mainly engaged the students who had their camera on. However, several students said that they turned off their cameras during the lectures because the session was being recorded. Another stated that having the camera on was particularly useful when having discussions in digital groups. The students who participated in the survey wished for more recorded lectures, indicating that their lecturers did not do this often.

One of the informants assumed that she would have turned off the camera when recording the lecture, and she thought she had not contributed much. She would have to consider whether a question was ‘stupid’ before asking it, and probably she had not asked any questions at all. She thought this was due to habit, and she indicated that one might get used to being recorded. That is, if recording had been the norm and she had become accustomed to it, it would have been easier to relate to.

All of the informants agreed that presentations with audio were useful, as the material could be repeated by rewinding to the desired location. They also reported that it sometimes took a while for the teachers to post such files, even though the students found these learning resources very useful.

They noticed an increased attendance rate among their peers in the online lectures, which they perceived as positive. The reason for the increased attendance, they believed, was that many students have to make a long trip to attend class, and the threshold for participating had become lower now that all teaching was online. This was supported by the qualitative results from the questionnaire, where a student said, ‘I commute several hours per school day to get to and from school, so I feel I have more time to work with school now that the lecture is online’.

However, one of the informants pointed out that it is important for students to be able to talk to each other when the lecturer is not present, that group activities should be arranged and that they should be provided with opportunities for voluntary meetings on campus in their spare time. One of the informants believed it to be important that the students themselves have a responsibility to address the learning environment and initiate meetings in both academic and social arenas. One felt that it was not desirable that the university was responsible for social contact between peers. It was suggested that time could be set aside, for example, after teaching, so that only students could talk together. It was expressed that in order to preserve social aspects in digital teaching and learning, the first meeting should be on campus. A mentor scheme was suggested, where former students could give tips and advice on how to function as a ‘digital student’.

#### Digital group work

The students expressed that they mainly collaborated well in digital groups (breakout rooms). Communication usually worked well with both the teacher and peers in these digital rooms. Nevertheless, some students reported that group work was not effective when it was carried out in ‘breakout rooms’. The students felt that the allocated time for group work was too short for collaboration, and some of the time was spent on technical challenges. There were also some students who withdrew from the group work, which the respondents believed was because some were shy. One student said that discussions during group work paid off and that communication worked well, but it was a pity that so few students participated. Getting to know the others in the group well was also deemed to be important for the level of collaboration and professional discussions. The students did not like to be randomly assigned into groups. However, they expressed that it would be advantageous to plan for more group work in smaller groups.

Another positive effect of online teaching the students highlighted was the increased amount of written feedback from lecturers on work submitted voluntarily. The students perceived that this was offered as a compensation for shorter teaching sessions.

One of the respondents thought that it was important to socially interact with peers and missed having lunch with fellow students. Others felt that there had not been many social gatherings in the group previously, and so they did not experience the absence of fellow students as a great loss. They also pointed out that students who had met each other physically at an earlier time had a different starting point in online meetings and for online education. One student stated, ‘Getting to know new peers digitally feels weird’. Furthermore, one of the informants pointed out that most people have a general need for physical contact, and that touching and eye-to-eye contact is important.

#### Motivation

Some of the students were more motivated to participate in online learning activities, yet it was perceived to require greater effort to stay motivated and ‘in the course’. Some students work alongside their studies and thus do not attend classes, and others have children who must be tended to. Some indicated that student response systems such as Mentimeter, Quizlet, Padlet, Kahoot! and the use of polls was motivating factors, but it depended on the context in which they were used. Some of the students reported that they especially liked Kahoot, but it was important that the use of such response systems was done in a structured way. They expressed that they liked the teaching programme, which consisted of an introductory video and teaching in which the basics were presented, followed by group work and finally teaching, where the teacher went more in depth. This approach made it easier to follow the teaching and to ask questions.

The students said it was good for motivation when an overview of the course content was published, as it contributed to predictability and more people participate when they know what is planned.

Nevertheless, the qualitative results from the questionnaire indicated that it was difficult to get an overview of everything that needed to be done. It could be challenging to concentrate and have self-discipline due to many distractions, which reduced the students’ motivation. Several students expressed that they felt alone in their studies, and it was difficult to feel alone with the responsibility for learning the curriculum. One student wrote that there was considerable uncertainty, which negatively affected concentration, and that the COVID-19 crises was a difficult time for everyone.

## Discussion

Overall, these students were satisfied with the ad hoc online teaching after the lockdown, although they experienced self-perceived reduced learning outcomes compared to the pre-pandemic situation. It appears that they adapted quickly to the new situation, but they also reported difficulties with the transition to new teaching methods. Based on both the surveys and interviews, the most pressing concerns among students were a lack of social interaction, housing situations that were unsuitable for home office purposes, including insufficient data bandwidth, and a sense of reduced motivation and effort. PHN is a small sized education which enables close contact between educators and students. The low student volume might explain why the dropout rate from the bachelor and master programs remained unchanged compared to that in previous years.

Receiving teaching, supervision, exams and assessments solely through online solutions was a new experience for these students. Apart from a 15-credit mandatory bachelor course offered as hybrid learning (7), traditional teaching methods still dominated the bachelor and master study programmes of PHN in winter 2020. Importantly, the students evaluated the ad hoc solutions offered during the chaotic spring of 2020 rather than a well-planned, high-quality online education using student-active methods [5]. Teachers switched to online teaching without any time to learn the technology, or standard quality online teaching practices [4]. They had many years of experience teaching in -person, and they had arranged their lessons and interactive elements around this mode of learning. Alternatively, they had very little experience teaching online. The students’ experiences in these online learning environments, which were thrown together at the last minute, are not necessarily indicative of students’ experiences in a quality online course based on principles from Quality Matters online education [19].

Although the students reported reduced learning outcomes after 12 weeks dominated by synchronous live-streamed lectures lasting for 30–45 minutes on Zoom, they had positive attitudes toward use of digital learning materials and tools in future online courses. For asynchronous lectures, the rule of thumb in online education is less than 10–15 minutes [19]. Although lectures of 45 minute duration is far beyond what is recommended for digital teaching [19], the students responded based on their recent experiences where many teachers, for reasons of feasibility, conducted their planned on-campus lectures digitally shortly after lockdown. Some of the students also reported that they especially liked Kahoot, however, since we wanted to keep the research questionnaire short, we did not ask more in detail for concrete digital tools. A pre-corona study from OsloMet reported that physiotherapy students’ attitudes toward a flipped classroom intervention were mainly positive, although the academic outcomes from the final exam were similar to those in previous years [20]. Further, in a recent large-scale pre-COVID-19 blended learning interprofessional course conducted a few weeks ahead of the lockdown, first-year bachelor’s students at OsloMet reported positive perceptions of the blended learning approach, using only short video clips (less than 10 minutes) [21]. Approximately 3/4 of the students in that study disagreed that virtual group discussions resulted in better learning outcomes than face-to-face group discussions. The present data do not conflict with the findings from that larger-scale study.

The students expressed in various ways that online teaching with a lack of social interaction leads to worse learning outcomes and lower levels of motivation and well-being. Concerns about lack of face-to-face contact may have been aggravated by the stressful situation, and contentment with teaching methods would likely improve if teachers had been able to integrate the appropriate elements in a fully digitalized course. Face-to-face interactions provide the foundation for social communication, the lack of which can be viewed as a critical disadvantage of online learning [5]. Face-to-face training may be particular crucial for candidates expected to have communication skills, such as nutritionists [11,12,22–24]. The ad hoc solutions for teaching offered during the 2020 spring term were thus not in agreement with the suggested conceptual dimensions, which allow students to expand their knowledge beyond the intended learning outcome established by the teacher: motivation and attention [5].

The students expressed concerns that are common in traditional in‐class teaching as well, and such issues should not be overlooked in online teaching [25,26]: insufficient pre‐class study preparation, limited participation and inadequate depth in class discussions. Quality of education lies in the knowledge, skills and expertise that are conveyed as well as in the manner in which they are communicated and learned [7,26]. In different ways, the students’ responses revolved around central quality aspects, such as learning objectives, content, programme design, adaptation, teaching, work methods, supervision and forms of assessment [7]. These findings are in agreement with other studies on COVID‐19 and education [4,25,27].

The students stated that they received insufficient information about the exams. This is understandable because staff initially did not know how the different exams would be digitally transformed in spring term 2020. Asked about exam preferences students said that they preferred longer written exams at home, over old campus-style exams, with short timelines, adapted to an online format. They also preferred multi-day written home exams over potential alternatives such as video or podcasts, which none of them had tried before. It should be noted that they had limited experience with digital options. Student-produced podcast and video have been used as formative assessment forms at our university [14], but to lesser extent as formative assessment forms. The preference for written home exams over digital options was thus likely influenced by student’s familiarity with the former since no exams during this time-period were in the form of podcast or video. Feedback and guidance from academic staff have been found to be key aspects of study quality, and good feedback contributes to increased motivation and improved learning outcomes (6). Exam uncertainty causes undue stress, and thus a key recommendation during the transition to online learning is to ensure that all information about exams is communicated to the students clearly and in a timely manner [27].

‘Black screens’ do not necessarily reflect individuals lack of motivation and attention or embarrassment, but they may reflect a lack of digital training among freshmen or technical issues, such as poor bandwidth. Broadband bandwidth overload issues and a lack of suitable equipment will probably not be significant problems in Norway in the future. The students suggested that both flipped classrooms and live streaming should be used in future online courses. Flipping the classroom [9] ahead of live streaming, with the possibility for the students to write down questions during the live streaming or afterward in a seminar, increases flexibility. Asynchronous tools may be utilised to support students to work at different times. We cannot overlook the possibility that new students might have needs that differ from those of senior students in terms of getting accustomed to online education. Nevertheless, our date indicates that clarification of expectations constitutes an important success criteria for online teaching, especially when it comes to group work and formative and summative assessment [4,27].

The closure of campus may have unknown implications for society in both the short and long term [28–30], including impacts on educational quality and the mental health of students and academic staff [31]. If students are unable to study effectively for some unknown reason, it will make online learning ineffective, regardless of educational quality. The situation after the lockdown in Norway was confusing, and many students lost their jobs and moved back in with their parents [4]. We did not collect person-sensitive data, and thus we know little about these students’ circumstances. The dropout rate remained nearly unchanged among these students as compared to previous years. Being a small-sized education, the staff were able to follow-up each student individually using digital videoconference tools, such as Zoom and Teams. In the future, more sustainable approaches should be developed, for example, by increasing peer-to-peer interactions and through mentoring programs [1]. Reducing dropout and increasing completion rates was a strategic goal for higher education before the lockdown [29], and we do not know the impact of the lockdown on future dropout and completion rates. The high dropout rate from Massive Open Online Courses (MOOCs) has been a major concern of researchers and educators over the years [32]. Although some universities worldwide had already started offering MOOC-based undergraduate degrees before the COVID-19 pandemic [32], most MOOCs do not lead to degrees. The online courses offered in spring 2020 after the lockdown were mandatory courses leading to degrees, and thus they were not directly comparable to the voluntary MOOCs. However, such issues are premature for consideration in the present study. OsloMet is currently participating both in the future ‘The COVID-19 Multi-Country Student Well-being Study’[33] and the ‘Corona and Campus’ study [34]. The ‘Corona and Campus’ study has secondary outcomes related to teaching satisfaction and learning outcomes, and such data will have the power to inform future decision-making [30]. However, the present data were collected shortly after the national lockdown due to the COVID-19 pandemic on aspects of digitalisation relevant to the (post)-pandemic situation.

### Strengths and weaknesses of the study

This study has several strengths. The most important strength is data collection shortly after a national lockdown due to the COVID-19 pandemic. The combined use of both quantitative and qualitative approaches enabled different perspectives to be captured and adds strength to the study. The triangulation allowed us to identify aspects more accurately and helped to offset the weaknesses of each approach alone. Group dynamics in focus group interviews can help bring out nuances in the data material beyond the answers to the predefined quantitative questions in the electronic questionnaires [17]. Another strength was the research team consisting of both external moderators providing objectivity, lack of vested interest and a fresh perspective, and internal evaluators who were familiar with the education and the students. One limitation is using a questionnaire which was not pre-tested or validated. However, due to time constraints shortly after campus lockdown following the COVID-19 outbreak, it was not possible to perform pre-testing or validation of the instruments used in the present study. Many of the necessary ad hoc changes to the course plans and exams (spring semester 2020) had yet to be made and decided upon when the present study was initiated, even when the first questionnaire was sent out before Easter 2020. The candidates actual achieved learning outcomes and working skills are unknown due to limited opportunities to monitor the quality of their work [4]. We do not consider it to be relevant to repeat the study, or reuse its instruments, since the acute phase after lockdown is over. PHN is a small-sized education, and the total number of students were only 79 individuals. The stress associated with the unprecedented situation may have contributed to a low response rate. Private circumstances such as poor internet connection, children at home, and lack of an adequate home office may also have contributed to a low response rate. A low response rate is also a limitation in studies performed in a normal situation [16]. We cannot rule out selection bias in the sample. The students who volunteered for the digital focus group interviews were positive and thorough. In particular, they seemed to reflect on a more general level, not restricted to their own personal situations. However, the range in age among the study participants was representative for the age range of all PHN students, and both bachelor and master students participated in the study. Data are collected from one single university, and the results might not be representative for large sized educations. Since the study is exploratory, we had not planned the data collection in order to test hypotheses. The study seeks to provide a snapshot in time of an evolving situation. Even with some limiting factors we believe the explorative study offers value since it provides a student perspective on an unprecedented black-swan event in higher education.

### Conclusions

Although they had little previous experience with online education, these students seemed to adapt quickly to the sudden shift to ad hoc online education due to the COVID-19 pandemic. The most pressing concerns among students were a lack of social interaction, a feeling of being alone in their studies, unfit housing situations for home office purposes, including insufficient data bandwidth, and a sense of reduced motivation and effort. Although our data indicate that face-to-face contact was greatly missed during this time-period, a thoroughly planned online course with numerous contact points between teachers and students would likely have been received more favorably. Finally, the students expressed that they wanted more structure in future digital courses. Due to the very unusual circumstances experienced both by students and teachers in the early stages of national lockdown in Norway, we are hesitant to conclude with regards to students preferences for future online courses.

## Supporting information

S1 File SPSS file questionnaire 1—please see line 154. (SAV)

S2 File SPSS file Norwegian questionnaire 1—please see line 154. (SAV)

S3 File SPSS file questionnaire 2—please see line 154. (SAV)

S4 File SPSS file Norwegian questionnaire 2—please see line 154. (SAV)

S5 File Structured interview guide–please see line 145. (DOCX)
