# Supplementary material for: Student’s experiences with online teaching following COVID-19 lockdown: A mixed methods explorative study
Source: PLoS One. 2021 Aug 31;16(8):e0250378. doi: 10.1371/journal.pone.0250378 (PMC8407578; doi:10.1371/journal.pone.0250378)
Supplement: S5 File — (DOCX) [file pone.0250378.s005.docx]

**INTERVIEW GUIDE**

**Help text for the moderators at the start of the interviews:** *«Welcome to digital focus group interview. Thank you for contributing. Due to the COVID-19 pandemic, OsloMet closed on 12 March 2020, and thus all teaching had to take place digitally for the rest of the semester. We would like to collect information about your experiences in relation to what has been as well as how you view future online teaching which is set to begin as early as this autumn. My name is…, and I am employed by OsloMet. My role here today is to lead the focus group interview. I will not participate in the conversation. Instead, I will listen and guide the conversation when needed. A focus group interview is a qualitative method, where one talks about a given topic. To provide written consent, please write “I agree” in the chat function without your name. I will save the chat. I will also take notes, but I will not record the conversation. Your participation is voluntary.*

**Part 1**

**RETROSPECTIVE QUESTIONS – Questions regarding the time after lockdown on 12 March 2020 (30 minutes)**

**Help text for the moderators***: It has been about 2 months since campus closure, and we would like to hear about your experiences in relation to the teaching offered in the time following this closure.*

**A) Introductory overarching question**

1. Tell us briefly about your experiences with digital teaching.

**B) Social aspects**

1. How has this time been for you compared to before lockdown? Did you face any special challenges? (Use a maximum of 5 minutes to answer this question, and do not provide personal sensitive data.)

2. What about the social consequences of the lockdown for you as compared to the time before the campus closed?

3. Have you noticed that some students do not meet in the digital rooms (or drop out) when you cannot meet them on campus? If so, has this resulted in any consequences for you?

**C) Teaching**

1. How do you find the different forms of digital teaching that you have experienced? (This question applies to both subject teaching and supervision.)

2. What do you think about the work requirements assigned to you and did you find them useful?

3. What is your experience of the exam modes that have been used and did you find them suitable?

4. What are your thoughts regarding communication with teachers and peers in the online courses?

5. How do you experience working in groups now compared to previously (e.g. in the breakout rooms)?

6. What do you think about your personal academic learning outcomes after the campus closed?

**D) Motivation**

1. What aspects have contributed to your increased/decreased motivation in connection to the assistance offered to you by the teachers?

2. How can different student response systems (e.g. Kahoot and Quizz) be used to motivate students?

3. Do you know of any digital tools that can increase student interaction?

**E) Technical aspects**

1. What do you think about “black screens”?

2. How have “black screens” affected you when the whole class attends and in breakout rooms?

3. What do you think about live digital readings (synchronous learning) compared to recordings (asynchronous learning)?

4. What are your thoughts on recorded lectures?

5. According to you, what is the optimal length of a (synchronous) digital lecture? (Short/long)

6. Have you experienced any limitations (e.g. technical) in relation to digital teaching?

**F) Concluding questions**

1. Have you experienced any special challenges and opportunities with regard to online teaching specifically in the field of nutrition this spring (i.e. that do not apply to other subject areas)?

2. Is there anything else you would like to add about the teaching you have experienced in the time after the campus was closed?

**Part 2 - THE FUTURE (approximately 30 minutes)**

**Help text for the moderators***: In this part of the interview, we will talk about what you think is important when creating fully digitally taught topics on nutrition at the Master’s level in the future.*

1. How can the practical aspects of teaching nutrition (cooking, self-registration of diet, etc.) be accomplished digitally?

2. With regard to learning design, what is the optimal distribution of digital lectures (recording/streaming), group work/breakout rooms, breaks, etc.?

3. How should students be assessed (with reference to course requirements and exams) in a future online Master’s course?

4. Which forms of digital teaching (both subject teaching and supervision) will benefit students the most in terms of learning?

5. Will mandatory digital presence add anything to the social context? If so, how?

6. Will mandatory digital presence play a role in academic learning outcomes? If so, how?

7. How can the student professional networks be improved?

8. What social interactions are likely to be useful in online study?

9. How can social interactions in groups be strengthened?

10. How can students be encouraged to participate digitally?

11. Should the university consider social aspects? In other words, should OsloMet initiate social interactions for online students?

12. Are there any other thoughts you would like to share?

**Thank you for participating!**
